# Nonverbal Immediacy Mediates the Relationship Between Interpersonal Motives and Belongingness

**DOI:** 10.3389/fsoc.2020.596429

**Published:** 2020-11-26

**Authors:** Eric Mayor

**Affiliations:** Department of Clinical Psychology and Epidemiology, University of Basel, Basel, Switzerland

**Keywords:** belongingness, social judgment, interpersonal motives, communion and agency, non-verbal immediacy

## Abstract

While belongingness is a predictor of mental and physical health, the lack of social bonds is an issue for many people in occidental countries. This issue calls for global and affordable solutions. In this study, we notably investigated (a) the presumed positive relationships between agentic and communal interactional motives and belongingness, and (b) the mediating role of self-reported non-verbal immediacy—an indicator of availability to interact—in these relationships. Cross-sectional and longitudinal data were collected by means of questionnaires to test these hypotheses (*N*_Crossectional_ = 344; *N*_Longitudinal_ = 126) using the General Belongingness Scale, the Non-verbal Immediacy Scale, and the Bem Sex Role Inventory. Results supported the hypotheses: Interpersonal motives and non-verbal immediacy are associated cross-sectionally to belongingness, non-verbal immediacy mediates the interpersonal motives—belongingness relationship and positive changes in non-verbal immediacy are also related to increased belongingness. Practical and research implications are discussed.

## Introduction

People crave to form and develop interpersonal relationships. Research notably attests to the pervasiveness of this need, of its emotional implications, and of the consequences of the lack of social bonds on mental and physical health (Baumeister and Leary, 1995; Ernst and Cacioppo, 2000; Cacioppo et al., 2002; Cohen, 2004; Hawkley and Cacioppo, 2010; Valcke et al., 2020). Oftentimes, lonely individuals are faced with the circular issue that the continued feeling of loneliness reduces the probability and expectation of forming gratifying social bonds in the future (Heinrich and Gullone, 2006; Sjåstad et al., 2020).

Belongingness refers to “stability, affective concern, and continuation into the foreseeable future” in individuals' social relationships (Baumeister and Leary, 1995, p. 500). There is suggestive evidence that lacking belongingness and loneliness are a common experience and a significant problem in Occidental countries (de Jong Gierveld et al., 2006; Heinrich and Gullone, 2006; Cacioppo and Patrick, 2008). Such widespread lack of social bonds is a serious issue, notably because of its health implications: Feelings of reduced belongingness have been associated with higher perceived stress (Cacioppo et al., 2006), higher risks of depression through a decrease in self-esteem (Lee and Robbins, 1998; Cacioppo et al., 2006; Heinrich and Gullone, 2006), and mental illness (Nuyen et al., 2020), and more extensively, psychological wellbeing (Valcke et al., 2020).

It is therefore important to promote individuals' belongingness on a large scale, rather than to focus merely on the few people who are the most in need, as is usually done in social skills training or social support groups (Cohen et al., 2000; Cruwys et al., 2014; Bandura et al., 2015). In this paper, we focus on this issue of social relevance from two interrelated perspectives: a social-motivational perspective, through the dimensions of agency and communion (how individuals relate to the social world); and a behavioral perspective, through perceived non-verbal immediacy (use of non-verbal signals of availability to interact)—which we will show, plays a mediating role in these relationships.

From a socio-motivational perspective, taking part in interactions and maintaining relationships is dependent upon interpersonal motives (Horowitz et al., 2006; Wong et al., 2013). Of course, people can be differently motivated to interact with one another. Agency and communion are the two main dimensions of interpersonal motives (Wiggins, 1991). Agency relates to a focus on self-interest, achievement, and personal influence, and communion to a focus on common interest and social participation (Horowitz et al., 2006).

From a behavioral perspective, attempts to enter interactions and create relationships are facilitated by displays of willingness to interact. A way to signal willingness to interact is non-verbal immediacy (Mehrabian, 1968; Burgoon et al., 1984). As we will see below, stronger interpersonal motives should relate to higher availability to interact (i.e., higher non-verbal immediacy).

In this paper, we examine whether agency and communion are related to general belongingness. If so, promoting agency and communion could foster belongingness and reduce loneliness. Using longitudinal analyses we will also show that an individual's perceived belongingness can be increased by non-verbal immediacy. Sustaining these positive changes might help foster belongingness at a large scale. In the next section, we discuss the relevant literature in more detail and present our hypotheses. But first, we present an illustrative example that will render the matter of this paper more concrete.

Ann, Bob, and Carl are former classmates who haven't seen each other for over a year. Carl, the youngest, just turned 21, and they have planned to party. In proximity to the bar entrance where they are all meeting, Ann sees Bob arriving. She smiles and waves while she greets him: “Hi Bob!” Bob thinks to himself that Ann is glad to see him, and he is glad to see her. He reciprocates the smiling, the gesturing, and the greeting: “Hi, Ann!” Then Carl arrives as Ann and Bob are talking. “Hi,” he says, still looking at his smartphone. Then, in response, Ann and Bob say, “Hi, Carl! Happy birthday!” Carl looks briefly at them and says, “Thanks.” He then focuses on his screen again, thinking to himself: “Why did I agree to this?” Ann and Bob continue their conversation while the three of them enter the bar.

In this epitome, Ann and Bob's exchange of displayed availability to interact (non-verbal immediacy) has strengthened their bond, at least momentarily. However, this display is lacking in Carl's behavior. If this is typical of Carl, Ann and Bob probably perceive him as not having much interest in spending time with people, or at least not with them. If this persists during the entire evening, Ann and Bob would probably prefer having their next drink together without Carl. This example reminds us that when it comes to maintaining relationships, our interpersonal motives and non-verbal display of psychological availability to others are as important as the words we speak. What's next clarifies both aspects.

## The Fundamental Interpersonal Motives

Agency and communion have been viewed as key dimensions of interpersonal motives (Wiggins, 1996; Horowitz et al., 2006; Locke, 2015). Agency refers to the degree of utilitarian conduct of individuals (Wiggins, 1991). Agentic individuals rationally pursue self-interests in their interactions, are more performance oriented, focus on their influence on others, and are more attracted to power (Wiggins, 1991; Horowitz et al., 2006; Mackinnon et al., 2013). Communal individuals pursue collective interests, are more interested in forming relationships and in intimacy, have generally a more prosocial conduct, and are more involved in the lives of others (Horowitz et al., 2006; Gebauer et al., 2014). Participation in communal activities increases connectedness at the level of the interaction (e.g., comparing feelings; Locke and Nekich, 2000).

Agentic and communal individuals are oriented to forming relationships for different motives, but interpersonal motives can be frustrated in the interaction (Shechtman and Horowitz, 2006; Locke, 2015; Wong et al., 2017). Belongingness not only depends on the strength of an individual's motivation to create social bonds but also on the reception by others of his/her interactional motives, as inferred from behavior (Galinsky et al., 2005). The creation and maintenance of such social bonds is facilitated by positive social judgment from others (Bell and Daly, 1984). Social judgment derives in part from the assessment by others of the probability of smooth social coordination with the target (higher probability is better; Galinsky et al., 2005). Agency and communion have also been frequently considered dimensions of social judgment (Fiske et al., 2007; Abele et al., 2008; Wojciszke et al., 2009; Abele and Wojciszke, 2014; Pietraszkiewicz et al., 2019; Hauke and Abele, 2020). Research shows that communion is a more important predictor of positive social judgment than agency, because people scoring high on communion are more likely to take into account interests of others, have higher degrees of perspective-taking, and are more prone to collaborate (Galinsky et al., 2005; Wojciszke and Abele, 2008; Laurent and Hodges, 2009; Wojciszke et al., 2009; Abele and Wojciszke, 2014); i.e., communal motives are generally evaluated more positively than agentic motives (McAdams et al., 1996; Cislak and Wojciszke, 2008; Uchronski, 2008; Wojciszke and Abele, 2008; Wojciszke et al., 2009; Abele and Wojciszke, 2014; Locke, 2015). Belongingness can be developed through strong (e.g., family, friends) and weak ties (e.g., acquaintances; Baumeister and Leary, 1995; Cohen, 2004), and social evaluations are more positively impacted by agency when evaluators are friends or family (strong ties), compared to when they are acquaintances or strangers (potential weak ties) (Wojciszke and Abele, 2008). Finally, agentic individuals are rated higher on social status and are more respected than communal individuals (Wojciszke et al., 2009; Carrier et al., 2014).

Research has focused on agentic and communal motives in the social world and on the social perception of agentic and communal individuals. Yet, the general relational outcomes of these dimensions, i.e., how they impact the individuals' social connectedness, have been overlooked.

## Non-Verbal Immediacy

We define non-verbal immediacy as the ensemble of non-verbal communicative behaviors that reflect psychological availability, communicate affiliation and preference, and result in perceived interpersonal closeness (Mehrabian, 1968; Burgoon et al., 1984). According to Expectancy Violation Theory (Burgoon, 1993, 2015; Burgoon and Hale, 1988), increasing such behaviors, even beyond social expectations (as long as appropriate), is associated with positive relational outcomes from the perspective of raters.

For instance, observers witnessing discrete interactions featuring high non-verbal immediacy rate the relationship between protagonists as more intimate and trustful than when immediacy is low (Burgoon et al., 1984). In daily-life contexts, such as interactions with students, subordinates, spouses, or during initial encounters, individuals' non-verbal immediacy is positively associated with likability ratings from others (Bell and Daly, 1984; Friedman et al., 1988; Fusani, 1994; Hinkle, 1999, 2001; Baringer and McCroskey, 2000) and with perception of relatedness with the target (Frymier et al., 2019).

As can be seen from these examples, the study of the impact of non-verbal immediacy has largely focused on interactional outcomes rated by others, rather than on the assessment of the individual who performs the assessed non-verbal immediate behaviors. While research has been silent on whether this impacts the person carrying on these behaviors beyond social judgment, the fact that people voluntarily increase non-verbal immediacy in order to increase liking certainly hints in that direction (Bell and Daly, 1984; Baringer and McCroskey, 2000).

One aim of the present study is to examine whether self-rated non-verbal immediacy indeed has positive implications for individuals in terms of belongingness, because the individual's assessment specifically matters when it comes to feelings of belongingness.

## Hypotheses

Research has shown that communion is related to the need to elicit feelings, whereas agency is linked with the need to pursue strivings (Aquino et al., 2016). Indeed, communal individuals are oriented to forming relationships and have a more intrinsic focus on interactions (e.g., Burgoon and Dillman, 1995; Wiggins, 1996), whereas agentic individuals not only strive for influence and power, but also for personal achievement (McAdams et al., 1996).

As such, agentic and communal interpersonal motives should both translate into the display of psychological availability to interact. We therefore hypothesize that communion (Hypothesis 1a) and agency (Hypothesis 1b) are positively related to self-reported non-verbal immediacy. Further indirect support for these hypotheses is provided by research showing that other-rated responsiveness (akin to communion) and assertiveness (akin to agency) are linked with other-rated non-verbal immediacy (Thomas et al., 1994).

Communal individuals are positively evaluated in terms of likability by more people (Wojciszke et al., 2009) and they focus on relationships intrinsically, rather than from a utilitarian (means to an end) perspective (Burgoon and Dillman, 1995; Wiggins, 1996). Agentic individuals are assessed as positively as communal individuals in strong ties, and they benefit from higher social status and respect which increases their potential for affiliation at large (Wojciszke et al., 2009; Carrier et al., 2014). Communion and agency are related to a comparable extent with higher other-rated trust for the target (Oleszkiewicz and Lachowicz-Tabaczek, 2016). We hypothesize that communion (Hypothesis 2a) and agency (Hypothesis 2b) are positively related with belongingness.

We pointed out that non-verbal immediacy signals willingness to interact (psychological availability), a prerequisite to most interactions. Research has notably shown that non-verbal immediacy is related to increased liking and other-perceived proximity to the target (e.g., Burgoon et al., 1984; Baringer and McCroskey, 2000). We therefore assume that non-verbal immediacy is a proximal predictor of belongingness, and hypothesize that self-reported non-verbal immediacy is positively related to belongingness (Hypothesis 3a). There is also evidence of reciprocity in non-verbal immediacy between co-interactants (Hale and Burgoon, 1984). Observer-rated relatedness is increased by non-verbal immediate behavior (Frymier et al., 2019) and individuals increase non-verbal immediacy to appear more likable (e.g., Bell and Daly, 1984). Further, feelings of belongingness can change rather rapidly (Baumeister et al., 1998; Locke and Nekich, 2000). We therefore hypothesize that positive changes in self-reported non-verbal immediacy are related to an increase in self-reported belongingness (Hypothesis 3b).

Past research has highlighted the role of partner-observable intervening variables in the effect of individual differences in interactional outcomes (e.g., White et al., 2004; Morry and Kito, 2009; Meier and Semmer, 2013). Interpersonal motives could affect belongingness, notably through observable behavior with interactional relevance. We hence hypothesize that the relationship between interpersonal motives and belongingness is mediated (see MacKinnon et al., 2007) by self-reported non-verbal immediacy: Self-reported non-verbal immediacy will mediate the relationship between communal interpersonal motives and belongingness (Hypothesis 4a), and self-reported non-verbal immediacy will mediate the relationship between agentic interpersonal motives and belongingness (Hypothesis 4b).

## Method

### Participants

A total of 356 participants took part in the first wave of data collection. We chose a sample of this magnitude based upon the recent literature (e.g., Study 1 in Abele and Wojciszke, 2007). Two participants did not indicate their gender, and 10 indicated an age below 18. These participants were excluded from the analyses. Our sample was balanced in terms of gender (53% female) and had an average age of 22.4 years (*SD* = 4). On average, 10 days after the first wave of data collection, respondents received a link to a second survey. Forty percent of participants lived with their parents. Four percent lived with other relatives, and 7% with their partner. Thirty percent of participants were sharing flats, whereas 16% lived alone and the rest had other living arrangements. Fifty-seven percent of the participants had an employment in parallel to their studies and only two participants had children. A total of 130 participants responded to the T2 questionnaire, on average 10 days after the T1 questionnaire. Four failed to answer to one or more items composing the scales used in this study. Therefore, their data at T2 could not be analyzed.

### Procedure

Participants were recruited on the campus of a Swiss university. Students who manifested an interest in the study personally gave their email address to a research assistant (826 addresses collected), through which they later received a personalized survey link to participate. Participants who completed both waves of questionnaires (T1 and T2) participated in a raffle for a tablet (value around 100 CHF, which translates to 102 USD).

### Measures

The following measures were collected along with others unrelated to the present report. Their role in the tests of the different hypotheses is indicated in Table 1.

**Table 1:** Variables, measurement occasions and roles in the tests of hypotheses.

	**Main study variables**					
	**Gender**	**Age**	**Communion**	**Agency**	**Non-verbal immediacy**	**Belongingness**
Measured at time 1	Yes	Yes	Yes	Yes	Yes	Yes
Measured at time 2	No	No	No	No	Yes	Yes
Role in H1a	Control	Control	IV	Control	DV (T1)	None
Role in H1b	Control	Control	Control	IV	DV (T1)	None
Role in H2a	Control	Control	IV	Control	None	DV (T1)
Role in H2b	Control	Control	Control	IV	None	DV (T1)
Role in H3a	Control	Control	Control	Control	IV (T1)	DV (T1)
Role in H3b	Control	Control	Control	Control	IV (T2); Control (T1)	DV (T2); Control (T1)
Role in H4a	Control	Control	IV	Control	Mediator	DV (T1)
Role in H4b	Control	Control	Control	IV	Mediator	DV (T1)

*IV, Independent variable; DV, Dependent Variable; T1, Time 1 measure; T2, Time 2 measure*.

Agency and communion were measured at T1 with a French translation of the Bem Sex Role Inventory (Bem, 1981) by Rogé 1992 (in Bouvard, 2009). This instrument features 20 items for Communion (femininity), 20 items for Agency (masculinity), and 20 filler items. Participants respond to this inventory on a 1 (Never) to 7 (Always) Lickert scale. The reliability of both scales was good (agency: *alpha* = 0.83, communion: *alpha* = 0.80). Example items are: “self-reliant,” “assertive” for Agency; and “gentle,” “compassionate” for Communion.

Non-verbal immediacy was assessed at T1 and T2 (within-subject) using a self-developed back-translation of the Non-verbal Immediacy Scale (NIS; Richmond et al., 2003). This 26-item measure is scored on a 5-point Lickert scale (from 1 = Never, to 5 = Very often). A sample item is: “I use my hands and arms to gesture while talking to people.” Cronbach's *alpha* was adequate in both questionnaires (all *alphas* > 0.89).

Belongingness was measured at T1 and T2 (within-subject) using a self-developed back-translation of the General Belongingness Scale (Malone et al., 2012). In other words, participants completed the measure twice. This 12-item scale is rated on a 7 point Lickert scale (from 1 = strongly disagree to 7 = strongly agree; all alphas > 0.88). An example item is: “When I am with other people, I feel included.”

Each scale was computed by averaging the items of which it is composed.

### Data Analysis

Process is a macro allowing for the test of different types of hypotheses which involve mediation, moderation, and conditional process analyses (Hayes, 2018). We used Process to generate the regression models required for the test of our hypotheses (except H3b) by relying upon the output of the mediation model: test of the effect of the independent variable on the mediator (see Model 1 in **Table 3**), the test of the effect of the independent variable on the dependent variable without the inclusion of the mediator as a predictor (total effect; see Model 2A), and test of the effect of the independent variable on the dependent variable with the inclusion of the mediator as a predictor (direct effect; see Model 2B). Additionally, the output for the mediation model provides tests of the indirect effects through bootstrapping and the Sobel *z* test (Hayes, 2018). Gender and age were included as control variables in the analyses as women and men differ in agency and communion (Prentice and Carranza, 2002), and age plays a role in social connectedness (Heinrich and Gullone, 2006). Because some authors have criticized the sole presentation of results of models including control variables (e.g., Spector and Brannick, 2011), we also include results for models without control variables. As indicated below, we draw the same conclusions from results of such unadjusted models as from models including statistical controls.

We used the data from participants who answered all items at T1 and T2 for the test of Hypothesis 3b, relying on a standard longitudinal regression model. Change in Belongingness results from the inclusion of Belongingness at T1 as a predictor of Belongingness at T2 (the dependent variable) because variance in Belongingness at T2 due to Belongingness at T1 is thereby partialled out. In other words, T2 scores are adjusted for T1 scores (Dalecki and Willits, 1991). Change in Non-verbal immediacy is modeled through the inclusion of Non-verbal immediacy at T1 as a covariate (thereby partialing out its explained variance on the dependent variable) and Non-verbal immediacy at T2 as a predictor of Belongingness at T2. The effect of change in Non-verbal immediacy from T1 to T2 is therefore represented by the coefficient for Non-verbal immediacy at T2. Gender, Age, Agency, and Communion were included in the model as control variables.

## Results

Participants who answered to the T1 questionnaire and those who answered both questionnaires did not differ with regards to any of the variables of interest in this study (all *t*s <1, all *p*-values > 0.3), and were similar in age, *t*(341) = 1.70, *p* = 0.096. A proportion test shows these participants did not differ with regards to gender either (T2 = 59% female, *z* = −1.1471, *p* = 0.250). There were no significant mean differences in neither non-verbal immediacy nor belongingness between measurement occasions (respectively *t*(125) = −0.356, *p* = 0.722; and *t*(123) = −0.368, *p* = 0.713).

Table 2 presents the descriptive statistics and correlations and Table 3 presents the results for hypotheses H1a, H1b, H2a, H2b, and H3b, in the traditional order of a mediation test (Baron and Kenny, 1986). Results in Table 3 were produced by Process (Hayes, 2018). Models displayed on this table include gender and age as covariates, but results hold when they are not included (see below).

**Table 2:** Correlation table and descriptive statistics.

	**M1**	**SD1**	**M2**	**SD2**	**1**.	**2**.	**3**.	**4**.	**5**.	**6**.	**7**.
1. Gender (Male = 1)	0.470	NA	0.41	—	—	0.139	0.280	−0.349	−0.230	−0.129	
2. Age	22.4	2.911	22.06	2.441	−0.039	—	0.062	−0.075	−0.054	−0.015	
3. Agency	4.459	0.683	4.474	0.717	0.280	−0.086	—	−0.109	0.212	0.174	
4. Communion	4.938	0.580	4.976	0.608	−0.256	−0.009	−0.096	—	0.350	0.154	
5. Immediacy T1	3.970	0.560	3.993	0.591	−0.250	−0.007	0.170	0.401	—	0.395	
6. Belongingness T1	4.81	0.812	4.010	0.610	−0.091	−0.017	0.113	0.213	0.504	—	
7. Immediacy T2	NA	NA	4.846	0.785	−0.221	−0.002	0.187	0.345	0.881	0.599	—
8. Belongingness T2	NA	NA	4.882	0.836	−0.089	0.050	0.140	0.210	0.516	0.885	0.623

*Correlations for the respondents to the first questionnaire are depicted above the diagonal (values higher than 0.106 are significant for an alpha threshold of 0.05). Correlations for the respondents to both questionnaires are depicted below the diagonal (values higher than 0.18 are significant for an alpha threshold of 0.05)*.

**Table 3:** Cross-sectional regression analyses *(N* = 344).

	**Model 1. DV: Immediacy T1**						**Model 2A. DV: Belongingness**						**Model 2B. DV: Belongingness**					
	***B***	***SE***	***t***	***p***	**LLCI**	**ULCI**	***B***	***SE***	***t***	***p***	**LLCI**	**ULCI**	***B***	***SE***	***t***	***p***	**LLCI**	**ULCI**
Constant	1.492	0.389	3.839	<0.001	0.727	2.257	2.819	0.592	4.757	<0.001	1.653	3.984	2.819	0.593	4.757	<0.001	1.653	3.398
Gender (Male =1)	−0.236	0.062	−3.806	<0.001	−0.357	−0.114	−0.242	0.094	−2.560	0.011	−0.427	−0.056	−0.128	0.092	−1.399	0.163	−0.308	0.052
Age	−0.004	0.01	−0.439	0.661	−0.023	0.015	0.000	0.015	0.015	0.988	−0.029	0.029	0.002	0.014	0.161	0.872	−0.025	0.030
Agency	0.258	0.042	6.071	<0.001	0.174	0.341	0.273	0.065	4.218	<0.001	0.146	0.400	0.149	0.065	2.298	0.022	0.021	0.276
Communion	0.311	0.051	6.068	<0.001	0.21	0.411	0.179	0.078	2.291	0.023	0.025	0.332	0.029	0.078	0.373	0.709	−0.124	0.183
Immediacy													0.482	0.079	6.113	<0.001	0.327	0.637
	***R***	***R2***	***F***	***df1***	***df2***	***p***	***R***	***R2***	***F***	***df1***	***df2***	***p***	***R***	***R2***	***F***	***df1***	***df2***	***p***
	0.47	0.221	23.969	4	338	<0.001	0.281	0.079	7.222	4	338	<0.001	0.413	0.171	13.874	5	337	<0.001

### Fundamental Interpersonal Motives, Non-verbal Immediacy, and Belongingness

We hypothesized that Communion and Agency are positively related with Non-verbal immediacy (H1a and H1b). These hypotheses are confirmed, as can be seen in Table 3, Model 1. These relationships also hold when the control variables (age and gender) are not entered in the model–for communion: *B* = 0.246, *SE* = 0.074, *t* = 3.652, *p* = <0.001, 95% C.I. = 0.100–0.0.931; for Agency: *B* = 0.230, *SE* = 0.063, *t* = 3.652, *p* = <0.001, 95% C.I. = 0.106–0.353 (*R*^2^ = 0.061, *F*_(2, 340)_ = 10.98, *p* <0.001).

We hypothesized that Communion (H2a) and Agency (H2b) are positively related with Belongingness. The results support our hypotheses (Table 3, Model 2A): Both Agency and Communion predict Belongingness beyond the control variables (age and gender). We note the relationship between Belongingness and both dimensions also holds when no control variable is included in the model—for Communion: *B* = −0.246, *SE* = 0.074, *t* = −3.318, *p* = 0.002, 95% C.I. = 0.100–0.391; for Agency: *B* = 0.230, *SE* = 0063, *t* = −3.652, *p* <0.001; 95% C.I. = 0.106–0.353. In Model 2A, belongingness is lower in men.

We hypothesized that self-reported Non-verbal immediacy is related to Belongingness (H3). The results support this hypothesis, as seen in Model 2B (Table 3). This also holds also when Gender and Age are not included as covariates in the model (*B* = 0.504, *SE* = 0.077, *t* = 6.539, *p* <0.001, 95% C.I. = 0.352–0.655, *R*^2^ = 0.166, *F*_(3, 339)_ = 22.473, *p* <0.001). We note that Gender is no longer a significant predictor of Belongingness after the inclusion of Non-verbal immediacy as a predictor of Belongingness (see Model 2B). Gender differences in self-reported non-verbal immediacy might explain the difference between men and women in Belongingness.

We now turn to the test of our mediation hypotheses. According to hypothesis 4a, the relationship between Communion and Belongingness is mediated by Non-verbal immediacy. The three models displayed in Table 3 provide support for this hypothesis. We used Process (model 4; Hayes, 2018; relying on MEDMOD in R Hubona and Lim, 2014) to generate the tests of total, direct and indirect effects of Communion on Belongingness, and the Sobel test: Total effect = 0.179, *SE* = 0.078, *t* = 2.291, *p* = 0.023, 95% C.I: 0.025 – 0.332; Direct effect: 0.029, *SE* = 0.078, *t* = 0.373, *p* = 0.709, 95% C.I. = −0.124–0.183; Indirect effect: 0.15, Bootstrapped *SE* = 0.039, Bootstrapped 95% C.I. = 0.084–0.233; Sobel *z* = 4.278, *p* <0.001. As shown by the significance of the total effect and the indirect effect, and the non-significance of the direct effect, the relationship between Communion and Belongingness is completely mediated by Non-verbal immediacy. In other words, the mediation is full because the effect of independent variable Communion on the dependent variable Belongingness is not significant anymore when the mediator Non-verbal immediacy is included in the model, while the indirect effect of the independent variable on the dependent variable is significant. This supports our hypothesis.

We ran the test of our second mediation hypothesis using process again. The relationship between Agency and Belongingness is mediated by Non-verbal immediacy (H4b): Total effect = 0.273, *SE* = 0.065, *t* = 4.218, *p* <0.001, 95% C.I: 0.146–0.400; Direct effect: 0.149, *SE* = 0.065, *t* = 2.298, *p* = 0.022, 95% C.I. = −0.124–0.183; Indirect effect: 0.124, Bootstrapped *SE* = 0.031, Bootstrapped 95% C.I. = 0.071–0.196; Sobel *z* = 4.279, *p* <0.001. As shown by the significance of the total, indirect and direct effects, the relationship between Agency and Belongingness is partially mediated by Non-verbal immediacy. In other words, the mediation is only partial here because the effect of independent variable Agency on the dependent variable Belongingness is still significant when the mediator Non-verbal immediacy is included in the model, and the indirect effect of the independent variable on the dependent variable is significant. This supports our hypothesis only partially.

Very similar results are obtained when we do not include Gender and Age as control variables–for Communion: Total effect = 0.245, *SE* = 0.074, *t* = 3.318, *p* <0.001, 95% C.I: 0.100 – 0.391; Direct effect: 0.055, *SE* = 0.076, *t* = 0.073, *p* = 0.464, 95% C.I. = −0.093 – 0.204; Indirect effect = 0.190, Bootstrapped *SE* = 0.042, Bootstrapped 95% C.I. = 0.119 – 0.282; Sobel *z* = 4.953, *p* <0.001; for Agency: Total effect = 0.230, *SE* = 0.0063, *t* = 3.365, *p* <0.001, 95% C.I: 0.106 – 0.353; Direct effect: 0.121, *SE* = 0.062, *t* = 1.974, *p* = 0.049, 95% C.I. = 0.004–0.244; Indirect effect: 0.108, Bootstrapped *SE* = 0.029, Bootstrapped 95% C.I. = 0.058–0.174; Sobel *z* = 4.010, *p* <0.001.

We hypothesized that change in Non-verbal immediacy is related to change in Belongingness (H3b). We tested this hypothesis using the data from participants who answered all items at T1 and T2 relying on a longitudinal regression model (see Methods). Gender, Age, Agency, and Communion were included as control variables. Our hypothesis is confirmed: *B*_ImmT2_ = 0.291, *SE* = 0.134, *t* = 2.168, *p* = 0.032, 95% C.I. = 0.025–0.556 (overall *R*^2^ = 0.811, *F*_(7, 115)_ = 70.522, *p* <0.001). We note similar results hold when these four control variables are not included as covariates: *B*_ImmT2_ = 0.275, *SE* = 0.134, *t* = 2.060, *p* = 0.042, 95% C.I. = 0.011–0.540 (*R*^2^ = 0.8, *F*_(3, 119)_ = 158.245, *p* <0.001).

## Discussion

Hundreds of studies have documented the benefits of social bonds regarding psychosocial well-being and health, notably through social support (Cohen et al., 2000; Cohen, 2004). This literature, however, has overlooked the motivational and behavioral determinants of social bonds.

Agency and communion are central interpersonal motives and dimensions of social judgment (e.g., Horowitz et al., 2006; Abele and Wojciszke, 2007, 2014; Wojciszke and Abele, 2008). In the literature, the relationships between agency, communion or non-verbal immediacy, and discrete interpersonal outcomes have been investigated from the perspective of others (e.g., Wojciszke and Abele, 2008). It was therefore not known whether actors' own interpersonal motives or non-verbal immediacy are related or not to actors' perceived interpersonal outcomes. In this paper, we focused on the role of the two fundamental motivational dimensions (agency and communion) in the prediction of belongingness. Our results show that both dimensions are positively related to belongingness (H1a and H1b) and thereby confirm our hypotheses.

Another original contribution of this paper has been to investigate non-verbal immediacy as an element of the process by which agency and communion affects belongingness. We found that both communion and agency are related to self-reported non-verbal immediacy (H2a, H2b), that non-verbal immediacy significantly impacts belongingness (H3a), and that it mediates the relationship of communion with belongingness (significant indirect and total effects; H4a), as hypothesized; and partially mediates the relationship between agency and belongingness (significant indirect, direct, and total effects; H4b). We also found intra-individual differences in non-verbal immediacy (around 20%) between measurement occasions, but no significant overall mean differences. Some participants increased in immediacy while others decreased.

The support for hypotheses H1a to H2b highlight the role of interpersonal motivational dimensions in shaping individuals' relationship as suggested by the mentioned literature, including a recent model of agency and communion (Abele and Wojciszke, 2014). This model states that agency and communion play a different role in the perception of the actor (agency more important) and the perception of the observer (communion more important; also see Wojciszke and Abele, 2008). Yet, our results have shown that communion and agency do not differentially impact either belongingness or non-verbal immediacy. Only the tests of the mediation hypotheses have shown differentiated roles of communion and agency. Indeed, the relationship between agency and belongingness (partially mediated by non-verbal immediacy) is more complex than the relationship between communion and belongingness (completely mediated), as shown by the significant direct effect of agency on belongingness in our mediation test of H4b. In other words, the role of agency on belongingness goes beyond its association with non-verbal immediacy. This supports the claims of Abele and Wojciszke (2007, 2014) of a more important role of agency in the actor's perception of interpersonal outcomes.

This study has also shown that positive changes in self-rated nonverbal immediacy were related to positive changes in self-rated belongingness (H3b). This constitutes an important contribution to research on belongingness and research on non-verbal immediacy as it highlights the importance of focusing on temporal factors, which have often not been examined in these fields. First, research on belongingness has mostly focused on static individual factors such as personality (Seidman, 2013), passion (Stenseng et al., 2015), and humor (Satici, 2020). The non-verbal immediacy—belongingness relationship clearly highlights the dynamic nature of belongingness. Second, non-experimental research on non-verbal immediacy has mostly examined its effects on a diversity of outcomes through experimental manipulation or considering it as a disposition (for an exception, see Hale and Burgoon, 1984). Focusing on the malleability of non-verbal immediacy instead of its purported static nature could allow the development of a research program focusing on its development and thereby supporting its beneficial outcomes, such as liking and belongingness (see Practical Implications below).

### A Quick Note on Gender

Although not the focus of this study, we quickly comment on the role of gender. Women and men differ on the main variables in this study: Women are higher than men in non-verbal immediacy, belongingness (except when non-verbal immediacy is controlled), and communion. They are lower than men in agency. These two latter results are frequently reported in the literature, as our society still values differences in the social roles embraced by women and men, which foster stereotypes and behavior (Eagly and Steffen, 1984; Prentice and Carranza, 2002; Koenig and Eagly, 2014). As noted in the results section, the disappearance of the effect of gender on belongingness when non-verbal immediacy is included in the model hints at a mediating effect of the latter variable also with regards to the effect of gender.

### Further Studies and Limitations

This study has focused on the determinants of belongingness. As we mostly used a cross-sectional design, we cannot make definite causal claims from our findings. Although causal claims can never be proven, this issue can be improved through diary studies investigating discrete interactions (Spector and Meier, 2014). Taking into consideration the outcomes of discrete interactions in predicting fluctuations in belongingness might also allow a better understanding of the roles of agency and communion in belongingness, notably through the identification of moderators of the relationships discussed in this paper. Another related limitation of this paper is that it does not take into account the impact of contextual variations of interpersonal motives (e.g., people might behave different interactionally at work than they do during their leisure time; Nezlek et al., 2007) on belongingness. As pointed out by Burgoon and Dillman (1995), some non-verbal immediacy behaviors can be the expression of different postures in the interaction, and are interpreted in context (e.g., while talking or while listening); for instance, gazing can be part of agentic conduct (dominance) as much communal conduct (affiliation), but in both case it signals psychological availability to interact. Does belongingness evolve differently depending on how others interpret these behaviors? Examining discrete interaction could help clarify all these points.

Another limitation of our study relates to the practical implications we propose below: Individuals could increase agentic and communal behaviors in order to increase belongingness, but this does not take into account the potential impact of behaving in ways that go against stereotypes. The literature on agency and communion as dimensions of social judgment has repeatedly shown the backlash against counter-stereotypical individuals in social evaluations (e.g., Rudman and Fairchild, 2004; Moss-Racusin et al., 2010). Whether such backlash affects belongingness should be investigated. For instance, the finding that people deploy much effort to redress misclassification of their identity (as a member of another group, Prewitt-Freilino and Bosson, 2008) shows they are well aware of interactional repercussions of stereotypes. Further, circumstances can interact with stereotypes as research has shown that the context impacts how agentic or communal individuals are perceived (Faniko et al., 2017). This too should be investigated, notably because our findings show that the relationship of agentic motives with belongingness is not fully mediated by non-verbal immediacy and might more strongly relate to social judgment.

Our study focused on non-verbal immediacy, but verbal immediacy is also an important predictor of interactional outcomes (Wiener and Mehrabian, 1968). For instance, an applicant's response latency, signaling higher or lower affiliation with the interviewer, can make the difference between getting a job or not (Brosy et al., 2016).

### Practical Implications

One avenue to improve belongingness might be for individuals to embrace agentic and communal behaviors. Although agentic and communal behaviors are of course related to interpersonal motives, they can also be voluntarily increased. Positive outcomes from individuals might go beyond increased belongingness. Buchanan and Bardi (2015) examined whether well-being is increased as a function of individuals' communion and agency in values, behaviors, or the fit of communal and agentic values and behaviors. They found that it is the behaviors that drive the positive effect.

As mentioned above, our results also show that there is malleability in non-verbal immediacy and that such changes can increase or decrease belongingness. This suggests that people scoring low on belongingness could build upon natural changes in non-verbal immediacy to strengthen their perceived belongingness. We didn't find mean differences between measurement occasions in non-verbal immediacy (some individuals increased, others decreased). Hence, to build up on these changes might require a voluntary focus on the performance of non-verbal immediate behaviors. Research has shown that non-verbal conduct can be practiced and learned (Riggio, 1986, 2006), and that it is easier to voluntarily increase non-verbal cues than to reduce them (De Paulo, 1992). Maintaining positive changes in non-verbal immediacy should not be too difficult for individuals, but of course, voluntarily increasing non-verbal immediate behavior requires an effort from the people who are low on non-verbal immediacy because they have not yet built this habit.

Findings of our study clearly point in the direction of considering high(er) non-verbal immediacy as a type of mental and physical health behavior. An efficient way to communicate such advice could be to rely on communication campaigns.

## Conclusion

The pervasiveness of lacking social bonds is an important social issue, as these bonds are beneficial for individual outcomes, such as well-being, mental and physical health, and longevity (e.g., Baumeister and Leary, 1995; Cacioppo et al., 2002). Our study has shown the importance of both communion and agency in the prediction of belongingness, and the mediating role of non-verbal immediacy in these relationships. As such, our study might be part of an answer to the issue of chronic loneliness (Hawkley and Cacioppo, 2010). Finally, our findings relate to the established relationships of agency and communion with other personal outcomes, such as well-being, stress reactions, and psychological and mental health (Helgeson, 1994; Lefkowitz and Zeldow, 2006; Sarrasin et al., 2014; Mayor, 2015), as such relationships might in part be mediated by belongingness. Further studies of these relationships might be beneficial for a healthier society.

## Data Availability Statement

The raw data supporting the conclusions of this article will be made available by the authors, without undue reservation.

## Ethics Statement

Ethical review and approval was not required for the study on human participants in accordance with the local legislation and institutional requirements. The patients/participants provided their written informed consent to participate in this study.

## Author Contributions

The author confirms being the sole contributor of this work and has approved it for publication.

## Conflict of Interest

The author declares that the research was conducted in the absence of any commercial or financial relationships that could be construed as a potential conflict of interest.
